# Unmet needs about iron deficiency in peritoneal dialysis: a Delphi consensus panel

**DOI:** 10.1186/s12882-022-02969-3

**Published:** 2022-10-20

**Authors:** Sandro Mazzaferro, Silvia D’Alonzo, Massimo Morosetti

**Affiliations:** 1 Department of Cardiovascular, Respiratory, Nephrologic and Geriatric Sciences, Sapienza University of Rome, Rome, Italy; 2 Department of Medical and Surgical Sciences, Nephrology Unit, Fondazione Policlinico Universitario “A. Gemelli” Istituto Di Ricovero E Cura a Carattere Scientifico, Rome, Italy; 3 Nephrology Unit, Giovambattista Grassi Hospital, via Giancarlo Passeroni 28, 00122 Rome, Italy

**Keywords:** Iron deficiency, Ferric carboxymaltose, Peritoneal dialysis, Chronic kidney disease, Anaemia

## Abstract

**Background:**

Anaemia and iron deficiency (ID) are common in chronic kidney disease (CKD) patients and related to outcomes. There is growing interest about the role of iron supplementation in CKD, particularly ferric carboxymaltose (FCM), also in relation to the use of erythropoiesis stimulating agents (ESAs). Despite a greater knowledge on ID management in patients receiving haemodialysis, a paucity of data exists about peritoneal dialysis (PD). Furthermore, the aim of this paper is to provide the results of a nationwide Italian survey about ID in PD using the Delphi method.

**Methods:**

A list of 16 statements (48 items) was developed about four main topics: (1) approach to iron therapy in PD; (2) management experience about iron therapy in PD; (3) ESA and iron in PD; (4) pharmacoeconomic impact. Using the Delphi methodology, the survey was distributed online to 36 Italian nephrologists with expertise in PD, who rated their level of agreement with each item on a 5-point Likert scale. Consensus was predefined as more than 66% of the panel agreeing/disagreeing with any given statement.

**Results:**

Twenty-five experts (70%) answered the survey. 35 items (73%) achieved a consensus (8 negative and 27 positive). In particular, the diagnosis of ID is widely known, but some doubts exist about how frequently test it. The use of I.V. iron seems to be routinary and can save money reducing the administration of ESAs. However, internal protocols are welcome.

**Conclusions:**

Expert PD nephrologists know well the problem of ID and feel the necessity of shared protocols to optimize the iron therapy and consequently the use of ESAs.

## Background

Anaemia is commonly encountered in chronic kidney disease (CKD) and is associated with reduced quality of life and increased cardiovascular disease, hospitalizations, cognitive impairment, and mortality [1]. In this context anaemia is a multifactorial process due to relative erythropoietin (EPO) deficiency, uremic-induced inhibitors of erythropoiesis, shortened erythrocyte survival, and disordered iron homeostasis [2, 3]. Iron deficiency (ID) is defined as either a true paucity of iron stores (absolute ID) or as a relative deficiency (functional) in which the patient exhibits an impaired iron release from body stores that is unable to meet the demand for erythropoiesis (also called reticuloendothelial cell iron blockade) [3, 4]. There is growing interest about the role of iron supplementation in CKD, particularly ferric carboxymaltose (FCM), also in relation to the use of erythropoiesis stimulating agents (ESAs) [5]. Indeed, ESA treatment results in a substantial increase in the iron demand for erythropoiesis [6, 7], and about 90% of ESA-treated patients require iron supplementation to sustain an optimal haematological response to ESAs [8, 9]. Moreover, despite a greater knowledge on ID management in patients receiving haemodialysis, a paucity of data and no international guidelines exist about peritoneal dialysis (PD). Also the Kidney Disease: Improving Global Outcomes (KDIGO) controversies paper about optimal anaemia management does not cite specific doubts about PD [10]. A specific necessity of having dedicated recommendations is growing, not borrowed from general CKD guidelines or haemodialysis indications. Furthermore, the aim of this paper is to provide the results of a nationwide survey about ID in PD using the Delphi method.

## Methods

This study was conducted in compliance with the Declaration of Helsinki. The Delphi method is a structured technique aimed at obtaining by repeated rounds of questionnaires a consensus opinion from a panel of experts in areas wherein evidence is scarce, and opinion is important [11–13]. The process has been structured into four phases. The survey was developed by a panel of 3 nephrologists, identified as key opinion leaders (KOLs) in this field in the Lazio region, Italy. The KOLs identified 16 statements (divided into 48 items) with a major need of clarification and debate, focused on the management of ID in PD patients (Tables 1, 2, 3 and 4). After approval by 5 external validators, who tested its understandability and clarity, the questionnaire was distributed to 36 expert nephrologists (panellists) via an online platform. The panellists were clinicians with solid experience in the field of PD, selected throughout the Country among public hospitals, no more than two per centre. In some countries nurses manage anaemia in this population and therefore make decisions about iron management, but this is not the case in Italy, so nurses were not administered the questionnaire.

**Table 1:** Approach to iron therapy in PD (topic 1)

**Statement 1**					
	**1**	**2**	**3**	**4**	**5**
**1.1 I retain that iron deficiency must be searched in peritoneal dialysis patients only when anaemia is present (defined accordingto current guidelines)**	**80%**		**20%**		
	*6*	*14*	*2*	*1*	*2*
**Statement 2**					
I retain that a correct approach to the diagnosis of iron deficiency in peritoneal dialysis involves thedetermination of:	**1**	**2**	**3**	**4**	**5**
2.1 Ferritine and TSAT	**8%**		**92%**		
	*0*	*2*	*2*	*2*	*19*
2.2 Sideraemia	**32%**		**68%**		
	*4*	*4*	*5*	*4*	*8*
2.3 Ferritine	**28%**		**72%**		
	*2*	*5*	*4*	*4*	*10*
2.4 MCV—Percentage of hypochromic erythrocytes	**20%**		**80%**		
	*0*	*5*	*10*	*5*	*5*
2.5 TSAT	**20%**		**80%**		
	*1*	*4*	*4*	*6*	*10*
**Statement 3**					
**I retain that the threshold values to recognize iron deficiency in peritoneal dialysis patients are:**	**1**	**2**	**3**	**4**	**5**
3.1 Ferritine < 100	**24%**		**76%**		
	*1*	*5*	*2*	*2*	*15*
3.2 Ferritine < 200	**24%**		**76%**		
	*1*	*5*	*7*	*9*	*3*
3.3 Ferritine < 300	**68%**		**32%**		
	*3*	*14*	*7*	*1*	*0*
3.4 TSAT < 20%	**8%**		**92%**		
	*0*	*2*	*2*	*5*	*16*
3.5 Sideraemia < normal reference values	**44%**		**56%**		
	*5*	*4*	*6*	*4*	*6*
**Statement 4**					
**I retain that:**	**1**	**2**	**3**	**4**	**5**
4.1 An internal protocol should exist about iron administration in every peritoneal dialysis centre	**10%**		**90%**		
	*1*	*3*	*5*	*8*	*8*
4.2 The *ars medica* should be followed	**48%**		**52%**		
	*3*	*9*	*6*	*1*	*6*
**Statement 5**					
**I retain that the frequency of tests to diagnose and follow-up iron deficiency should be:**	**1**	**2**	**3**	**4**	**5**
5.1 Monthly	**60%**		**40%**		
	*6*	*9*	*6*	*2*	*2*
5.2 Bi-monthly	**40%**		**60%**		
	*5*	*5*	*4*	*6*	*5*
5.3 Quarterly	**36%**		**64%**		
	*4*	*5*	*5*	*6*	*5*
5.4 Only in relation to clinical needs	**80%**		**20%**		
	*12*	*8*	*2*	*1*	*2*
5.5 Accordingto local / regional rules *(if they respect clinical needs)*	**88%**		**12%**		
	*10*	*12*	*3*	*0*	*0*

**Table 2:** Management experience about iron therapy in PD (topic 2)

**Statement 6**						
**I principally treat my patient in PD with iron deficiency by:**	**1**	**2**	**3**		**4**	**5**
6.1 Oral iron	**8%**		**92%**			
	*1*	*1*	*12*		*7*	*4*
6.2 I.V. iron	**16%**		**84%**			
	*1*	*3*	*4*		*10*	*7*
**Statement 7**						
**In relation to the treatment strategy, I retain that**	**1**	**2**	**3**		**4**	**5**
7.1 It should be modulated on correction targets set for every patient	**0%**		**100%**			
	*0*	*0*	*6*		*8*	*11*
7.2 It should be a schema “low doses—high frequency”	**90%**		**10%**			
	*3*	*16*	*3*		*3*	*0*
7.3 It should be a schema “high doses at a low frequency”	**12%**		**88%**			
	*1*	*2*	*6*		*12*	*4*
**Statement 8**						
**I retain that target values in the peritoneal dialysis patient are:**	**1**	**2**		**3**	**4**	**5**
8.1 TSAT not inferior to 20% and not superior to 40%	**4%**			**96%**		
	*0*	*1*		*4*	*8*	*12*
8.2 TSAT not inferior to 10% and not superior to 30%	**64%**			**36%**		
	*7*	*9*		*4*	*5*	*0*
8.3 TSAT not inferior to 30% and not superior to 50%	**52%**			**48%**		
	*3*	*10*		*6*	*4*	*2*
8.4 Ferritine inferior to 500	**20%**			**80%**		
	*0*	*5*		*7*	*6*	*7*
8.5 Ferritine inferior to 500	**44%**			**56%**		
	*8*	*3*		*4*	*5*	*5*
**Statement 9**						
**Referring to the fact that I.V. therapeutic strategies now available facilitate the treatment of iron deficiency in peritoneal dialysis 1 retain that**	**1**	**2**		**3**	**4**	**5**
9.1 The currently adopted approach is adequate for the management of the majority of patients in PD	**20%**			**80%**		
	*0*	*5*		*7*	*8*	*5*
9.2 The strategies that could be applied with new molecules let both logistic issues and clinical response improve	**4%**			**96%**		
	*0*	*1*		*1*	*9*	*14*
9.3 The improvement is solely about clinical response	**60%**			**40%**		
	*3*	*12*		*6*	*4*	*0*
9.4 The improvement is solely about safety	**60%**			**40%**		
	*3*	*12*		*7*	*2*	*1*
9.5 The improvement is solely about logistic issues	**60%**			**40%**		
	*3*	*12*		*5*	*5*	*0*
**Statement 10**						
**I retain that the patient with heart failure and iron deficiency in PD:**	**1**	**2**		**3**	**4**	**5**
10.1 Should be treated as every other patient	**72%**			**28%**		
	*7*	*11*		*2*	*4*	*1*
10.2 Needs a greater attention as regards iron deficiency and a more complete and rapid correction	**8%**			**92%**		
	*0*	*2*		*2*	*12*	*9*
10.3 Only cardiologic indications should be followed	**92%**			**8%**		
	*9*	*14*		*1*	*1*	*0*

**Table 3:** ESA and iron in PD (topic 3)

**Statement 11**							
	**1**	**2**		**3**	**4**	**5**	
**11.1 I retain that a greater attention iron deficiency issues can impact on the decision to start using ESA**	**0%**			**100%**			
	*0*	*0*		*4*	*7*	*14*	
**Statement 12**							
	**1**	**2**		**3**	**4**	**5**	
**12.1 I retain to take care of iron deficiency issues since 1 need to reduce the use of ESA**	**20%**			**80%**			
	*1*	*4*		*9*	*6*	*5*	
**Statement 13**							
	**1**	**2**		**3**	**4**		**5**
**13.1 I retain that the reduction of the use of ESA (without interrupting I.V. iron) is functional to the achievement of certain levels of haemoglobin**	**4%**			**96%**			
	*0*	*1*		*5*	*15*		*4*
**Statement 14**							
	**1**	**2**		**3**	**4**	**5**	
**14.1 I retain that I.V. iron should be interrupted at the achievement of certain levels of hemoglobin and only afterwards ESAs should be reduced**	**60%**			**40%**			
	*3*	*12*		*5*	*5*	*0*	
**Statement 15**							
**In peritoneal dialysis Hb levels I retain satisfactory are:**	**1**	**2**	**3**		**4**	**5**	
15.1 10 -12	**20%**		**80%**				
	*0*	*5*	*9*		*5*	*6*	
15.2 11–12	**4%**		**96%**				
	*0*	*1*	*4*		*9*	*11*	
15.3 10,5–11,5	**20%**		**80%**				
	*2*	*3*	*9*		*11*	*0*

**Table 4:** Pharmacoeconomic impact (topic 4)

**Statement 16**					
**Referring to the use of I.V. iron and to the costs optimization for the treatment of anaemia in the patient in peritoneal dialysis, I retain:**	**1**	**2**	**3**	**4**	**5**
16.1 That an evaluation should be performed	**8%**		**92%**		
	*2*	*0*	*5*	*13*	*5*
16.2 That the use of ferric carboxymaltose let a reduction of the use of ESA which is able to overrule the greater cost of the drug itself	**8%**		**92%**		
	*0*	*2*	*3*	*9*	*11*
16.3 To observe an improvement in clinical outcomes but not in the use of ESA	**32%**		**68%**		
	*1*	*7*	*10*	*5*	*2*
16.4 That the hospital pharmacy does not consent the use of iron molecules at a higher cost	**68%**		***32%***		
	*5*	*12*	*4*	*4*	*0*
16.5 That pharmacoeconomic data now available do not let to clearly highlight an advantage among different molecules	**52%**		**48%**		
	*4*	*9*	*7*	*3*	*2*

The four main topics were: (1) approach to iron therapy in PD; (2) management experience about iron therapy in PD; (3) ESA and iron in PD; (4) pharmacoeconomic impact. Panellists were invited to express their level of agreement or disagreement on each item using a five-point Likert scale, scored from 1 to 5 (1, extremely disagree; 2, disagree; 3, agree; 4, mostly agree; and 5, extremely agree). Results were expressed as a percentage of respondents who scored each item as 1 or 2 (disagreement) or as 3, 4, or 5 (agreement). A positive consensus was reached in case of agreement > 66%, a negative consensus in case of disagreement > 66% while, when the sum for disagreement or agreement was below 66%, the consensus was not reached [12, 13].

Descriptive statistics were used to summarize the results.

## Results

**Fig. 1:**
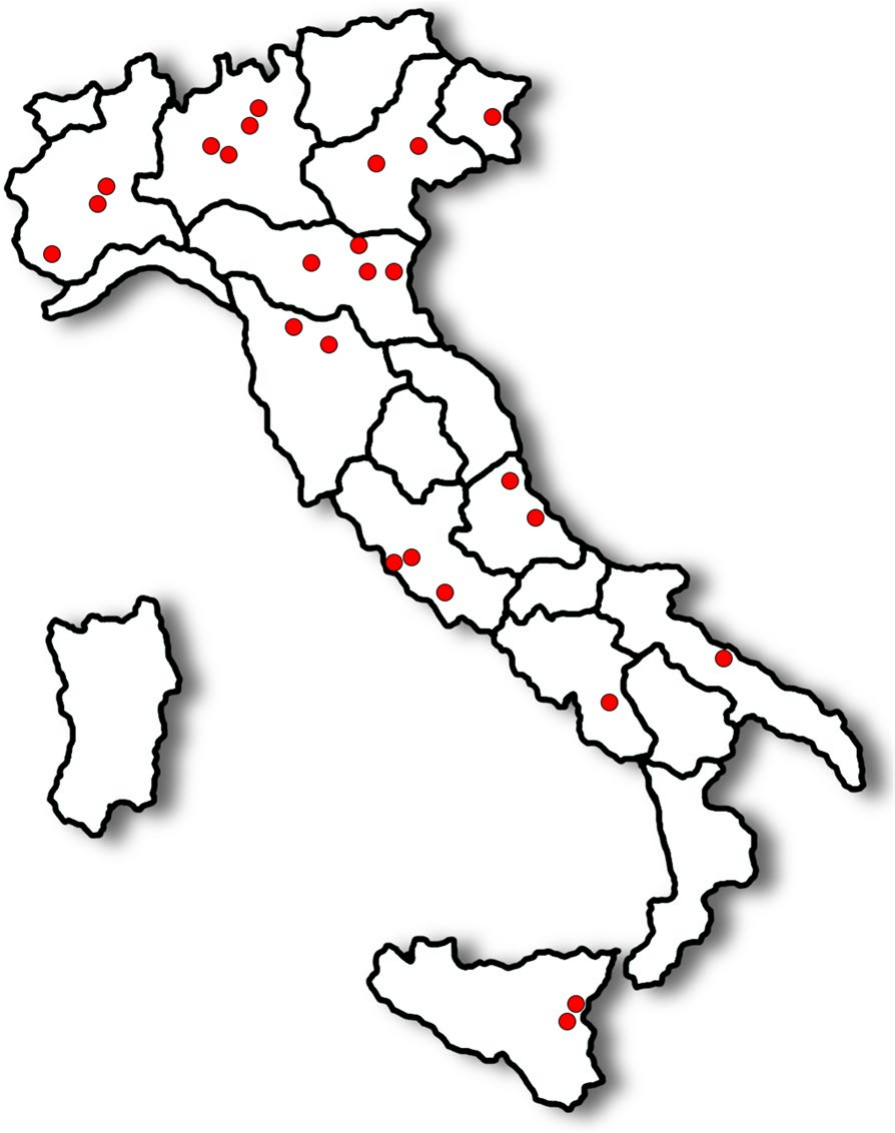
National distribution of respondents

The respondents were 25 out of 36 invited panellists (response rate 70%). Non respondents claimed lack of time or interest, as well as change of nephrology field. The national distribution of respondents is highlighted in Fig. 1. A total of 9 (36%) of the respondents were female. Overall, 35 (73%) items of the Delphi survey reached consensus, while no consensus was reached for 13 (27%) statements.

Tables 1, 2, 3 and 4 summarize the statements and presents the percentage of agreement/disagreement for each one based on the responses of the 25 panellists.

### Topic 1: approach to iron therapy in PD (Table 1)

The panel fully agreed in considering ID and anaemia two independent conditions (statement 1). In order to diagnose ID a positive consensus with the highest level of agreement was reached for the combination of ferritin and the percentage of transferrin saturation (TSAT); however, a positive consensus was reached also for the two single elements, as well as for iron and MCV (statement 2). Accordingly, the threshold values to diagnose ID were consensually set at ferritin < 100 ng/mL or < 200 ng/mL and at TSAT < 20%, while the panellists agreed not to consider ferritin < 300 ng/mL as a reference value. In addition, no consensus was found about the role of iron threshold (statement 3). The panel retained necessary to have a protocol for iron administration in PD, rather than operate according to the “ars medica” (statement 4). However, no consensus was reached about the frequency of the exams to monitor ID (with every 3 months being nearer to consensus), which should not be according to clinical need or local rules (statement 5).

### Topic 2: management experience about iron therapy in PD (Table 2)

The panellists agreed that the current route of iron supplementation in PD is either oral or intravenous, with a greater confidence with the last strategy (statement 6). They retained that the strategy pursued should be focused on individual targets and with a “high dose, low frequency” schema, instead of a more frequent and fractionated administration. (statement 7). The target values for iron therapy which reached a consensus were only TSAT between 20 and 40% and ferritin < 500 ng/mL, while no consensus was reached for the other options (statement 8). Despite the current approach is considered adequate in the majority of patients, the new molecules are retained to be able to improve both logistic and clinical aspects, while safety issues did not reach a consensus (statement 9). Regarding the PD patients with heart failure and ID, the nephrologists involved agreed not to treat them as the other patients without heart failure and not to pursue the cardiologist indications only, but they need a greater attention to ID and a more complete and rapid correction (statement 10).

### Topic 3: ESA and iron in PD (Table 3)

The panel agreed that a greater attention on ID can impact on the decision to start an ESA (statement 11) and on the reduction of their use (statement 12), when Hb target has been reached due to iron supplementation and ESA reduction (statement 13).

Accordingly, no consensus was expressed about the interruption of intravenous iron before ESA reduction when the haemoglobin target is reached (statement 14). Regarding the haemoglobin target, the panel agreed for different values, all between 10 and 12 (statement 15).

### Topic 4: pharmacoeconomic impact (Table 4)

The panellists retained that an evaluation about cost optimization in anaemia treatment in PD should be performed, since the higher cost of FCM is compensated by a reduced administration of ESAs. However the nephrologists expressed a contradictory belief about the reduction in the use of ESAs.

A negative consensus was reached about the forbidden permission to adopt high-cost iron by the hospital pharmacy.

Finally, the panel did not express a consensus about the absence of net pharmacoeconomic data favouring some iron formulations (statement 16), suggesting that the research to date is promising and quite reassuring.

## Discussion

This Delphi panel suggests that ID is a recognized problem also in PD patients. In particular, ID is independent from anaemia and iron status must be investigated in all PD patients, as guidelines generally recommend in CKD patients [1]. Indeed, it is known than ID is related to outcomes in PD patients [14]. However, an universal recommendation about how to measure it and which thresholds consider is still lacking [5]. As the panel answered, the majority of guidelines suggest the combination of ferritin and TSAT as the best tools to assess ID, since ferritin alone can be falsely elevated by the inflammatory status, but also hypochromic red cells and iron are cited [1, 5, 15–18]. Most commonly, ID should be treated when ferritin < 100 ng/mL and TSAT < 20% [1, 5, 15–18]: these were the two items with the higher confidence from the panel. However, a ferritin threshold of 200 ng/mL is also considered in haemodialysis patients [19], with less conviction received by the panel. The role of iron itself as a laboratory test is not cited by guidelines about ID management in CKD. However, probably thanks to its availability in most laboratories and the consequent widespread report, no consensus was reached by the panellists about its use in ID. Furthermore, the need of an internal shared protocol is claimed by the majority of respondents, since the common sense in clinical practice is not a valid strategy for them. In fact, since 1996 a consensus paper was published in Taiwan to solve this issue [20]. On the other hand, a greater randomness is expressed by the panel about how frequently search for and follow-up ID. Indeed, guidelines suggest when to test anaemia, but not ID [1, 5, 15–18]. In particular, the panel was more prone to test every 2–3 months, approaching a consensus. On the contrary, a screening is considered mandatory.

The present Delphi consensus highlights the higher confidence of nephrologists about I.V. than oral iron, focused on tailored targets different from patient to patient. In particular, high dose-low frequency strategy is preferred, which is typical of FCM. In general, the panel felt adequate the currently adopted approach (i.e., high dose-low frequency) for the majority of PD patients, but new molecules such as FCM may improve the logistics and the clinical response. Indeed, this was already demonstrated by Portolés-Pérez et al. in a multicentre retrospective real world study enrolling 91 PD patients: FCM was effective, safe and easy to administer during routine clinical visits, letting 68.6% of patients achieving ferritin levels of 200–800 ng/mL, 78.4% TSAT > 20%, and 62.8% TSAT > 20% and ferritin > 200 ng/mL after 12 months [21]. Ferritin between 200 and 800 ng/mL and TSAT > 20% were considered for drug efficacy. However, a greater variability is present among guidelines in this field, in part caused by the different cut-offs for patients in haemodialysis [1, 5, 15–18]. TSAT > 20% and ferritin up to 800 ng/mL are now considered safe. The last threshold, however, is more recent and an upper value of 500 ng/mL is the most cited by guidelines [1, 5, 15–18], as reflected by the agreement received by the panel. This explains the doubts of the panellists about this topic. Vice versa, nephrologists demonstrated a greater knowledge of the most recent trials about heart failure and ID; in particular, FCM reduced rehospitalizations in patients with a recent acute heart failure event [22]. However, when such complex patients are in PD, the panellists felt that their iron management should be shared with cardiologists, instead demanded to them.

ESA administration is crucial for anaemic CKD patients to correct EPO deficiency. On the other hand, ESA treatment increases the iron demand for erythropoiesis [6, 7], and about 90% of ESA-treated patients require iron supplementation to sustain an optimal haematological response to ESAs [8, 9]. Since nephrologists have been using FCM, the relationship and cost-effectiveness of ESAs and FCM itself has become more complex. The panellists agreed that a correct use of FCM can normalize the iron status and probably reduce the waste of costly ESAs, overruling the initially higher cost of FCM itself than other compounds. To summarize, they felt that I.V. iron can be prioritized than ESAs to reach the target haemoglobin also in PD patients, as already suggested by guidelines [1, 5, 15–18]. In particular, physiologically when the target haemoglobin is reached with iron supplementation and ESA, the last should be reduced, instead of iron stopped as more frequently happens in clinical practice. In addition, as already reported, the iron status in partly independent from the haemoglobin level and should be investigated with different exams (i.e., ferritin and TSAT). Notably, the target haemoglobin varies among guidelines, as reflected by the answers of the panel: values between 10 and 12 g/dL are generally considered adequate in PD since they are more stable than in haemodialysis thanks to a constant volaemia.

Finally, regarding pharmacoeconomic aspect the panel revealed to be quite imprecise, with contrasting answers. While an evaluation of cost-efficacy of I.V. iron, particularly FCM, should be performed, reducing costs and improving clinical outcomes, The panel retained that at the moment the data are already promising and quite reassuring about an advantage among different iron formulations.

Notably, the reduced use of ESAs may overrule the initially higher cost of FCM, but this field should be better investigated.

To the best of our knowledge, this is the first consensus Delphi about a highly specific topic such as ID in PD patients. The board of clinicians felt the necessity to have dedicated recommendations, not borrowed from general CKD guidelines or haemodialysis indications. Indeed, the major strengths of this paper is the rigorous method on its basis and its novelty. In addition, the 25 panellists were chosen among active nephrologists expert in PD. This explains the notable knowledge about iron management to treat ID, probably mediated by the haemodialysis field. Indeed, pharmacoeconomic and organizational aspects are mostly unknown for this kind of clinicians. Finally, being the respondents active in PD centres, the national representativeness on this Delphi is high, since PD centres are not extensively diffuse throughout the Country (Fig. 1).

## Conclusions

In conclusion, expert PD nephrologists know well the problem of ID and feel the necessity of shared protocols to optimize the iron therapy and consequently the use of ESAs.

## Data Availability

All data generated or analysed during this study are included in this published article.

## Abbreviations

CKD: Chronic kidney disease
EPO: Erythropoietin
ID: Iron deficiency
FCM: Ferric carboxymaltose
ESAs: Erythropoiesis stimulating agents
PD: Peritoneal dialysis
KOLs: Key opinion leaders
TSAT: Transferrin saturation
